# A podiatry survey: how do we treat hallux valgus conservatively?

**DOI:** 10.1186/1757-1146-8-S2-O19

**Published:** 2015-09-22

**Authors:** Sheree Hurn, Bill Vicenzino, Michelle Smith

**Affiliations:** 1 School of Clinical Sciences, Queensland University of Technology, Kelvin Grove, QLD, 4059, Australia; 2 School of Health and Rehabilitation Sciences, The University of Queensland, St Lucia, 4072, Australia

**Keywords:** Hallux valgus, podiatry, treatment, survey

## Background

Hallux valgus (HV) is a prevalent foot deformity with limited research available to guide decisions regarding conservative management. Clinical guidelines are largely based on expert opinion with little evidence of what actually occurs in day-to-day practice. An increased understanding of non-surgical treatment options currently utilised by podiatrists is needed to potentially improve clinical practice, education and research. The aim of this survey was to explore current practice among Australian podiatrists treating HV in light of current clinical guidelines.

## Methods

An online survey specific to HV was developed based on focus group data previously collected. The survey was distributed to Australian podiatrists in mid 2013 via the professional association in each state (approximately 1900 members). Respondents indicated how they would manage HV across three patient groups: juveniles, adults, and older adults. Proportions were calculated to determine the most common responses and a descriptive analysis was performed in comparison with clinical guidelines and evidence.

## Results

Of the 210 survey respondents (11% of all surveyed), 65% (136) were female. 80% (168) of respondents worked solely in private practice. 159 complete survey responses were received for treatment of juveniles, 146 for adults and 141 for older adults. The most common response for management of HV across all age groups was “advice regarding different footwear”. Juveniles were likely to be offered pre-fabricated orthotic devices (67%) and muscle strengthening or retraining (51%). Exercises were less likely to be offered to adults (33%). Adults were commonly offered orthotic devices, either custom (75%) or prefabricated (54%), while older adults were most often treated using footwear modifications (59%) and padding (55%). These treatment recommendations are largely consistent with clinical guidelines, although only a small proportion of podiatrists reported recommending anti-inflammatory medications (17% in adults and 23% in older adults).

## Conclusions

Despite the lack of empirical evidence in this area, there are consistent trends regarding conservative management of HV among podiatrists in Australia, and these are largely in line with clinical guidelines based on expert opinion. Management strategies clearly differ across patient age groups. This study provides useful data that could inform policy, conservative management and future clinical trials.

